# Distribution of Bovine Mastitis Pathogens in Quarter Milk Samples from Bavaria, Southern Germany, between 2014 and 2023—A Retrospective Study

**DOI:** 10.3390/ani14172504

**Published:** 2024-08-29

**Authors:** Verena Bechtold, Wolfram Petzl, Reglindis Huber-Schlenstedt, Ulrike S. Sorge

**Affiliations:** 1 Department of Udder Health and Milk Quality, Bavarian Animal Health Services, 85586 Poing, Germany; reglindis.huber@tgd-bayern.de (R.H.-S.); ulrike.sorge@tgd-bayern.de (U.S.S.); 2 Clinic for Ruminants with Ambulatory and Herd Health Services, Centre for Clinical Veterinary Medicine, Ludwig Maximilians University Munich, 85764 Oberschleissheim, Germany; wpetzl@lmu.de

**Keywords:** bovine mastitis, mastitis pathogens, incidence, season

## Abstract

**Simple Summary:**

Bovine mastitis is the most common disease affecting the dairy industry and is mostly caused by intramammary infections (IMIs) due to mastitis pathogens. In this retrospective study we investigated the distribution of mastitis pathogens in all quarter milk samples (QMSs) submitted to the Bavarian Animal Health Service (TGD) in Southern Germany between 2014 and 2023. Overall, 19% of the QMSs contained mastitis pathogens and the most frequently isolated pathogens, in decreasing frequency, were non-aureus staphylococci (NAS), *Staphylococcus (S.) aureus*, *Streptococcus (Sc.) uberis*, and *Sc. dysgalactiae*. However, differences were found in the distribution of the mastitis pathogens depending on the mastitis status of the quarter from which the samples originated and the time of year.

**Abstract:**

The objective of this study was to investigate the distribution of mastitis pathogens in quarter milk samples (QMSs) submitted to the laboratory of the Bavarian Animal Health Service (TGD) between 2014 and 2023 in general, in relation to the clinical status of the quarters, and to analyze seasonal differences in the detection risk. Each QMS sent to the TGD during this period was analyzed and tested using the California Mastitis Test (CMT). Depending on the result, QMSs were classified as CMT-negative, subclinical, or clinical if the milk character showed abnormalities. Mastitis pathogens were detected in 19% of the QMSs. Non-aureus staphylococci (NAS) were the most common species isolated from the culture positive samples (30%), followed by *Staphylococcus (S.) aureus* (19%), *Streptococcus (Sc.) uberis* (19%), and *Sc. dysgalactiae* (9%). In culture-positive QMSs from CMT-negative and subclinically affected quarters, the most frequently isolated pathogens were NAS (44% and 27%, respectively), followed by *S. aureus* (25% and 17%, respectively) and *Sc. uberis* (8% and 22%, respectively). In QMSs from clinically affected quarters, the most frequently isolated pathogens were *Sc. uberis* (32%), *S. aureus* (13%), *Sc. dysgalactiae* (11%), and *Escherichia (E.) coli* (11%). The distribution of NAS and *Sc. uberis* increased throughout the study period, while that of *S. aureus* decreased. From June to October, QMSs from subclinically affected quarters increased and environmental pathogens, such as *Sc. uberis*, were detected more frequently. In conclusion, this study highlights the dynamic nature of the distribution of mastitis pathogens, influenced by mastitis status and seasonal factors. Environmental pathogens still play an important role, especially in clinical mastitis and seasonal dependency, with the number of positive samples continuing to increase. It is therefore essential to continue mastitis control measures and to regularly monitor the spread of mastitis pathogens in order to track trends and adapt targeted prevention measures.

## 1. Introduction

Bovine mastitis is the most common disease in the dairy industry, leading to significant economic losses due to reduced milk production and quality [1]. Apart from the economic consequences, mastitis also affects animal welfare and raises public health concerns due to the increased use of antibiotics [2]. Typically, bovine mastitis is caused by pathogens like staphylococci, streptococci, or coliform species that induce intramammary infections (IMIs) [3]. These IMIs can manifest either clinically, with typical signs of inflammation, or subclinically, without visible signs [4]. The pathogens that cause mastitis are traditionally classified into contagious and environmental, or alternatively major and minor pathogens. The first classification depends on the transmission of the causative pathogens. Contagious pathogens like *Staphylococcus (S.) aureus* and *Streptococcus (Sc.) agalactiae* are thought to spread predominantly via milk droplets among cows [5]. This usually happens during milking time, where the hands of the milker, towels, or the milking machine serve as a fomite for the transmission of contagious pathogens [6]. In contrast, environmental pathogens such as *Sc. uberis* and coliforms as *Escherichia (E.) coli* are commonly found in the cow’s environment, such as their bedding or lanes. From there, they can infect the udder [7]. Major pathogens often cause clinical mastitis and can remain in the udder for an extended period, whereas minor pathogens usually cause less severe reactions [8,9]. Historically, contagious pathogens such as *Sc. agalactiae* and *S. aureus* were the primary cause of mastitis [2]. In the 1960s, a five-point plan was developed by the National Institute for Research in Dairying, which was later expanded to a ten-point plan, that included practices such as teat dipping after milking, proper maintenance of milking equipment, and treating all cows with antibiotics at dry-off [2,10]. These measures led to a significant reduction in contagious mastitis pathogens and shifted the prevalence towards environmental pathogens [11]. Besides poor hygiene, environmental factors, such as hot and humid climate, can promote the growth of environmental mastitis pathogens [1]. For example, dairy cows are affected by heat stress in the warmer months, and a high temperature–humidity index may lead to a higher shedding of mastitis-causing pathogens [12]—even in moderate climates such as Germany’s.

In Germany, environmental pathogens, *Sc. uberis* in particular, have the highest prevalence and are responsible for the majority of clinical mastitis cases in Northern Germany [13]. This was also reported for Bavaria, where *Sc. uberis* also accounts for the majority of clinical mastitis cases [14]. Bavaria is a significant dairy-producing region in Germany, housing 30% of German dairy cows. The predominant breed is Simmental cows and the average herd size is 44 cows per herd [15,16]. This differs from other parts of Germany, e.g., eastern Germany, where the predominant breed is Holstein-Friesian and the average herd size is 197 cows [17]. Regional differences of dairy production may impact the distribution of mastitis pathogens. Therefore, it is important to monitor trends of those pathogens for specific regions over a longer period. This knowledge may directly impact management practices and preventive measures on dairy farms and thus plays an essential role in improving dairy cattle health. Therefore, the objectives of this study were to investigate the distribution of mastitis pathogens in quarter milk samples (QMSs) submitted to the laboratory of the Bavarian Animal Health Service (TGD) between 2014 and 2023 (a) in general, (b) in relation to the clinical status of the quarters from which the QMS originated, and (c) to analyze possible seasonal differences in the detection of mastitis pathogens.

## 2. Materials and Methods

This retrospective study included all quarter milk samples (QMSs) sent to the milk quality laboratory of the Bavarian Animal Health Service (TGD) between 2014 and 2023 (*n* = 3,886,162). The samples used in this study were collected for herd health management and diagnostic purposes. Institutional Animal Care and Use Committee approval was therefore not necessary.

Most QMSs were taken from whole herd screenings by TGD technicians (around 83% across the years). Those herd screenings were requested by the farmer or veterinarian, and generally, TGD technicians collected aseptic QMSs from all functioning quarters of lactating cows. This was carried out to check the intramammary infection status within herds, improve udder health, or even to help avoid a milk delivery ban in rare instances. Due to the small herd size (on average around 45 cows), all lactating cows were usually sampled to identify the predominant mastitis pathogen within the herd. In some cases, e.g., larger herds or follow-up sampling, a subset of cows might have been chosen for examination. The remaining QMSs (about 17% across the years) were submitted by farmers or veterinarians for individual case screenings, such as cows with clinical mastitis or before drying off. In Germany, usually, all quarters of a cow are sampled because billing is on a per cow (not sample) basis and the sample is collected in sterile 9 mL vials with boric acid. A semiquantitative analysis of somatic cells in milk of each QMS was evaluated directly on farm by TGD technicians or upon arrival at the milk laboratory using the California Mastitis Test (CMT). Samples were classified as negative (N) if the CMT showed a negative or as subclinical (S) if the CMT showed a positive result. Clinical mastitis (C) was diagnosed if the milk showed abnormal characteristics or if the cow exhibited other signs of clinical mastitis (e.g., swollen udder). This classification was determined either by technicians during on-farm sampling or through visual examination of the milk in the laboratory.

### 2.1. Bacteriological Analysis

All QMSs were culturally tested in the TGD milk laboratory according to the (at the time) current guidelines of the German Veterinary Association (DVG) (e.g., [18]). Each QMS was cultured on esculin blood agar based on Columbia agar with sheep’s blood additive using an inoculum volume of 0.01 mL or 0.05 mL for QMSs from clinically affected quarters. The plates were incubated aerobically at 36 ± 2 °C. Evaluations were performed after 18–24 h and 48 h of incubation. If it was not possible to evaluate the plates twice, they were only evaluated after 36 h of incubation. The determination of a positive result depended on the respective species in accordance with DVG guidelines.

The isolates were initially differentiated based on colony morphology, Gram stain, hemolysis, and hemotoxin zones. *S. aureus* was identified by colony morphology and hemolysis (clear zone of β-hemolysis). Clumping factor or coagulase were assessed only in isolates that did not exhibit a clear zone of β-hemolysis to distinguish them from non-aureus staphylococci (NAS). MALDI-TOF (microflex MALDI Biotyper, reference database V.3.3.1.0., Bruker Daltonik GmbH, Leipzig, Germany) was used for strains with unclear results or for further differentiation into individual species (e.g., *S. haemolyticus*, *S. chromogenes*, *S. epidermidis*). NAS were rarely further differentiated. To simplify matters, the identified staphylococcal species were divided into *S. aureus* and NAS.

Streptococcal strains were differentiated based on several criteria, including colony morphology, hemolysis pattern, esculin hydrolysis, CAMP (Christie–Atkins–Munch–Peterson) factor, and classification into Lancefield groups. To identify the according Lancefield group, a commercial test kit was used (Streptex^TM^ Acid Extraction KT/50TST, ZL59/R30951301, Thermo Scientific™, Waltham, MA, USA). Esculin-negative strains were further differentiated using the CAMP factor test, in which a β-hemolytic *S. aureus* strain was used. *Sc. agalactiae* was identified as esculin-negative and CAMP factor-positive, in contrast to *Sc. dysgalactiae*, which was identified as esculin-negative and CAMP factor-negative, belonging to Lancefield Group C. Strains of streptococci displaying significant β-hemolysis within Lancefield Group G were identified as *Sc. canis*. Esculin-positive streptococci were cultured on KAA-Agar (kanamycin–esculin–azide agar, Merck 1.05222.0500, Darmstadt, Germany), an in-house method with an agar selective for enterococci, and a disk test against penicillin (10 μg, Oxoid CT0043B, Thermo Scientific™, Waltham, MA, USA) and rifampicin (2 μg, Oxoid CT0078B, Thermo Scientific™, Waltham, MA, USA). Depending on the results of this method, strains could be classified into *Sc. uberis*, *Enterococcus (E.)* spp., or other esculin-positive streptococci. Using MALDI-TOF, species could be further differentiated but were here summarized as *Enterococcus* spp. (*E. faecium*, *E. faecalis*), *Lactococcus (L.)* spp. (*L. lactis*, *L. garvieae*), and other esculin-positive streptococci (if not applicable to either enterococci or lactococci). *Trueperella (T.) pyogenes* was identified on the basis of colony morphology, hemolysis pattern and, if necessary, microscopy.

Further differentiation by MALDI-TOF was carried out for all Gram-negative rod bacteria. Here, species were summarized as *E. coli*, *Serratia (Se.)* spp. (incl. *Se. marcescens*), and Gram-negative species (e.g., *Pseudomonas* spp., *Pasteurella* spp., *Proteus* spp., *Klebsiella* spp., coliforms, *Raoultella* spp.).

Other rarely detected species that were not applicable to the other groups were classified as “others” (e.g., *Nocardia* spp., *Listeria* spp., *Mycoplasma* spp.).

In the following text, the pathogens are assigned as follows: Environmental pathogens: *Sc. uberis*, *Sc. dysgalactiae*, *E. coli*, *T. pyogenes*, NAS, other esculin-positive streptococci, *Serratia* spp., and Gram-negative pathogens. Contagious pathogens: *S. aureus*, *Sc. agalactiae*, and *Sc. canis.*

### 2.2. Statistical Analysis

SAS 9.4 software (SAS Analytics Software Institute Inc. SAS Institute GmbH, Heidelberg, Germany) was used for the statistical analysis. PROC FREQ procedures were used to display the individual pathogens by year, month, and mastitis status. Chi-square test was used to compare prevalence of the different mastitis status, and according to sample origin (herd/individual). The Cochran–Armitage trend was used to assess a prevalence trend for each pathogen over the years and months. The graphics were created using Microsoft Excel 2010 (Microsoft Excel for Microsoft 365 MSO, Version 2308, Redmond, WA, USA) and α was set at 0.01.

## 3. Results

Between 2014 and 2023, 3,886,162 QMSs from 634,022 cows and 15,609 herds were analyzed. Of the total, 1.5% of QMSs were contaminated and therefore excluded. Mastitis pathogens were cultured in only 19% of the QMSs, meaning that 81% showed no growth. One pathogen was detected in 95.5% of the culture-positive QMSs and two pathogens were detected in 4.5%. Overall, 729,459 isolates of mastitis pathogens were included in this analysis.

Table 1 provides an overview of all QMSs included in this study. Of the total, 83% of QMSs came from herd samplings, while the remaining were sent in by farmers or veterinarians from individual case investigations. Herd screenings contributed 85% of the samples from CMT-negative quarters and 67% from subclinically affected quarters. In contrast, QMSs from clinically affected quarters were more likely to come from individual samplings (*p* < 0.01). Furthermore, there was a sharp increase in individual submissions from clinically affected quarters from an average of 40% (2014–2017) to an average of 55% (2018–2023, *p* < 0.01). As the majority of samples were from herd screenings, the majority of isolates were detected in samples from herd screenings. Only *E. coli* (68%) and *T. pyogenes* (57%) were found more frequently in samples from individual submissions than herd screenings (*p* < 0.01).

Considering culture-negative and culture-positive QMSs, the apparent prevalences of the most frequently isolated pathogens were as follows: NAS (5.7%), *S. aureus* (3.6%), *Sc. uberis* (3.6%), and *Sc. dysgalactiae* (1.6%).

**Figure 1:**
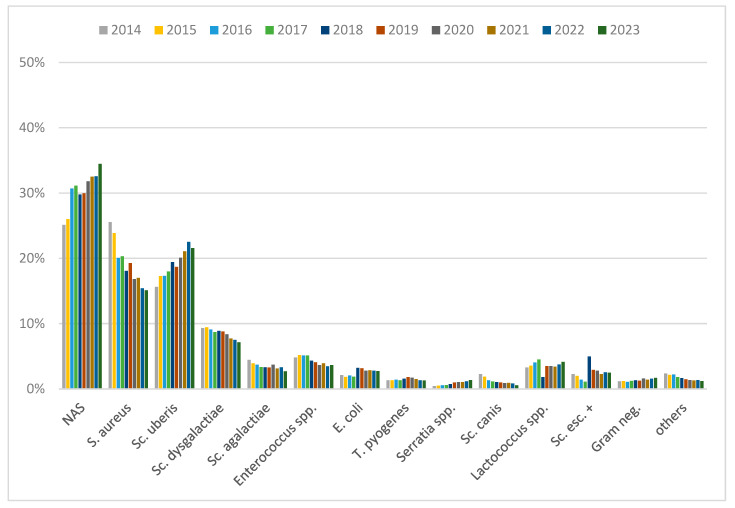
Distribution of mastitis pathogens in culture-positive samples per year. NAS = non-aureus staphylococci; S. = *Staphylococcus*; Sc. = *Streptococcus*; E. = *Escherichia*; T. = *Trueperella*; Sc. esc. + = other esculin-positive streptococci; Gram neg. = Gram negative pathogens.

Figure 1 shows the distribution of pathogens in culture-positive samples over the years. The most frequently isolated pathogens in culture-positive samples were NAS (30%). Though the vast majority of NAS (95%) were not further differentiated, the rest consisted of 2% *S. chromogenes*, 0.9% *S. epidermidis*, 0.8% *S. haemolyticus*, 0.5% *S. simulans*, 0.4% *S. xylosus*, and others less than 0.2%. The second most common pathogen in culture-positive samples was *S. aureus* (19%). The distribution of *S. aureus* isolates declined from 26% in 2014 to 15% in 2023 (*p* < 0.01). In contrast, NAS (25% to 34%) and *Sc. uberis* (16% to 22%) isolates increased over the whole study period (*p* < 0.01). An increase in *E. coli* isolates was observed from 2018 onwards (2014–2017: 2% to 2018–2023: 3%, *p* < 0.01).

### 3.1. Distribution in Dependence of Mastitis Status

**Figure 2:**
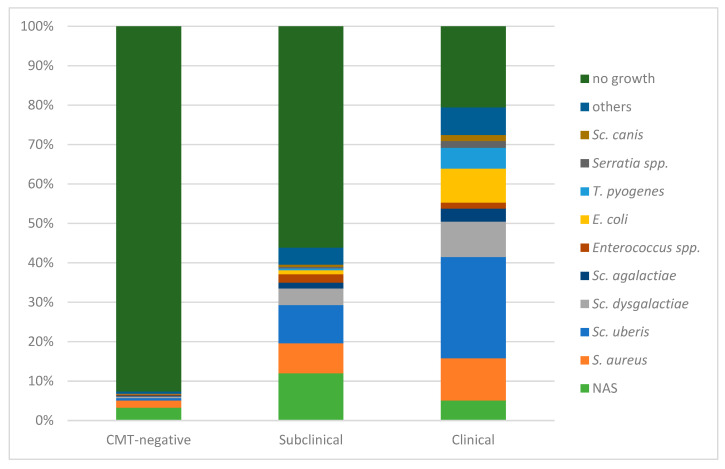
Distribution of no-growth samples and mastitis pathogens in CMT-negative, subclinically affected, and clinically affected quarters. NAS = non-aureus staphylococci; Sc. = *Streptococcus*; S. = *Staphylococcus*; E. = *Escherichia*; T. = *Trueperella*; all other detected species are included in “others”.

The distribution of mastitis pathogens shifted depending on the mastitis status of the udder (Figure 2). Thus, pathogens were more likely cultured from QMSs from clinically affected quarters (*p* < 0.01), compared to CMT-negative or subclinically affected quarters. When considering all QMSs, the apparent prevalences of the most commonly detected mastitis pathogens in samples from CMT-negative quarters were as follows: NAS (3%), *S. aureus* (2%), *Sc. uberis* (0.6%), and *Sc. dysgalactiae* (0.4%). In QMSs from subclinically affected quarters, the apparent prevalences were NAS (12%), *Sc. uberis* (10%), *S. aureus* (8%), and *Sc. dysgalactiae* (4%). In QMSs from clinically affected quarters, the apparent prevalences were *Sc. uberis* (26%), *S. aureus* (11%), *Sc. dysgalactiae* (9%), and *E. coli* (9%).

**Figure 3:**
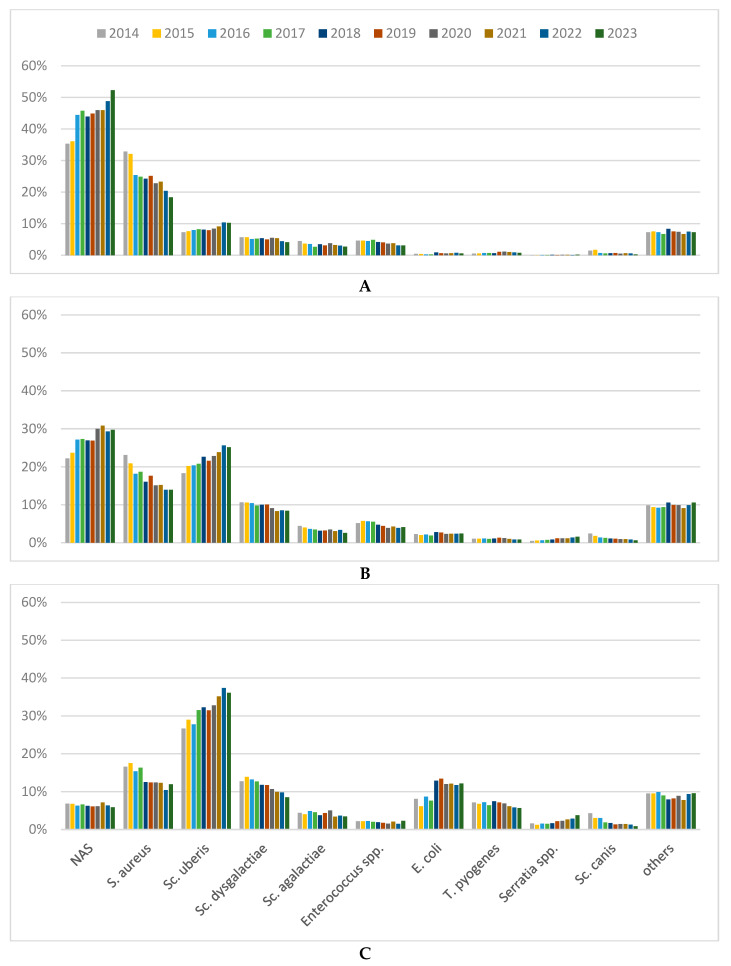
Distribution of mastitis pathogens from culture-positive samples from CMT-negative (**A**), subclinically affected (**B**), and clinically affected (**C**) quarters per year. NAS = non-aureus staphylococci; S. = *Staphylococcus*; Sc. = *Streptococcus*; E. = *Escherichia*; T. = *Trueperella*; all other detected species are included in “others”.

Figure 3A–C shows the annual distribution of mastitis pathogens from culture-positive samples for each mastitis status. While NAS were the most common pathogens in QMSs from CMT-negative (44%) and subclinically affected quarters (27%), they were only isolated in 6% of QMSs from clinically affected quarters. The distribution of NAS from CMT-negative and subclinically affected quarters increased over the study period (Figure 3A,B, *p* < 0.01), while no trend was observed for the distribution of NAS from clinically affected quarters (Figure 3C, *p* = 0.29); 43% of NAS from clinically affected quarters were undifferentiated; the differentiated NAS included *S. chromogens* (16%), *S. epidermidis* (11%), *S. haemolyticus* (10%), *S. simluans* (8%), *S. xylosus* (3%), *S. sciuri* (3%), and others at less than 2.5% each. Similarly, *S. aureus* was detected more frequently in QMS from CMT-negative (25%) and subclinically affected quarters (17%) than in clinically affected quarters (13%). Overall, *S. aureus* isolates from all three mastitis classifications showed a decline in their detection in culture-positive samples during the study period (Figure 3A–C, *p* < 0.01, respectively), though the distribution of *S. aureus* isolates from clinically affected quarters remained at a constant level from 2018 onwards (*p* = 0.02).

In contrast, *Sc. uberis*, *Sc. dysgalactiae*, *E. coli*, *T. pyogenes*, and *Serratia* spp. were more common in QMSs from clinically affected quarters (Figure 3C, *p* < 0.01, respectively). *Sc. uberis* was the most frequently isolated pathogen in QMSs from clinically affected quarters (32%) and was detected in only 8% of QMSs from culture-positive and CMT-negative quarters. *Sc. dysgalactiae* was the third most frequent pathogen in clinically affected QMSs (11%)—after *Sc. uberis* and *S. aureus*. *E. coli* had 10% higher detection in samples from clinically affected quarters compared to CMT-negative quarters (1%, *p* < 0.01). *T. pyogenes* and *Serratia* spp. showed a 5% and 2% higher distribution, respectively, in QMSs from clinically affected quarters than in QMSs from CMT-negative quarters (*p* < 0.01, respectively). *Sc. uberis* isolates showed an increase across all three mastitis classifications (*p* < 0.01, respectively), while *Sc. dysgalactiae* isolates showed a decrease across all three mastitis classifications (*p* < 0.01, respectively). *E. coli* showed a clear increase in culture-positive QMSs from clinically affected quarters (from 8% in 2014 to 12% in 2023, *p* < 0.01), with a sudden increase from 2017 (8%) to 2018 (13%, *p* < 0.01). *T. pyogenes* isolates showed no trend regardless of mastitis status (*p* > 0.08, respectively). In contrast, *Serratia* spp. showed a steady increase in QMSs from clinically affected quarters (2% to 4%, *p* < 0.01).

### 3.2. Seasonal Distribution of Mastitis Pathogens

Differences in the mastitis status of culture-positive QMSs were found depending on season. In the warmer months, from June to October, the frequency of CMT-negative samples decreased (on average from 30% to 26%, *p* < 0.01) and the frequency of subclinical samples increased (on average from 62% to 66%, *p* < 0.01). In contrast, the proportion of QMSs from clinically affected quarters remained constant at 8% across all months.

**Figure 4:**
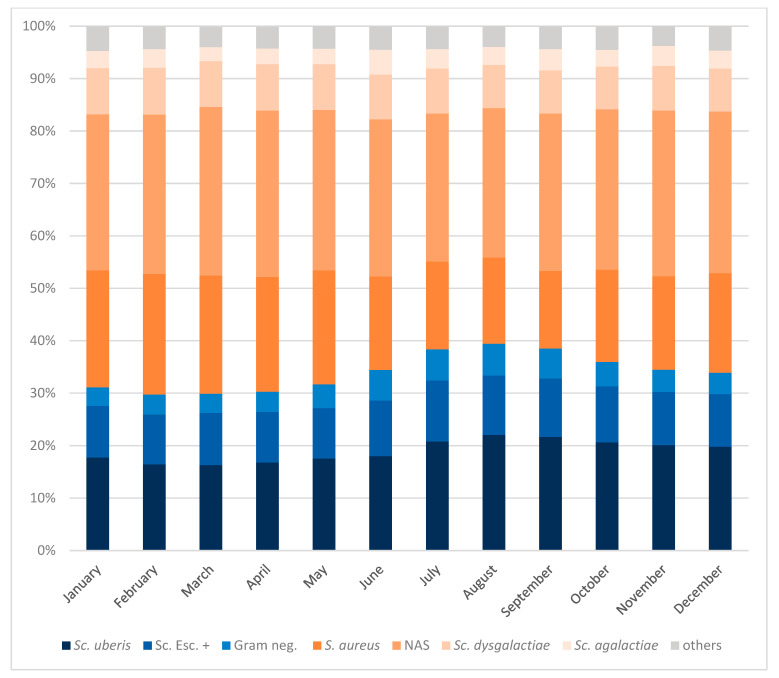
Distribution of mastitis pathogens in seasonal comparison per month in culture-positive samples. Sc. = *Streptococcus*; Sc. esc. + = other esculin-positive streptococci (incl. *Lactococcus* spp. and *Enterococcus* spp.); Gram neg. = Gram-negative pathogens (incl. *E. coli* and *Serratia* spp.); S. = *Staphylococcus*; NAS = non-aureus staphylococci. Others: all others, incl. *Sc. canis* and *T. pyogenes*.

Figure 4 provides an overview of the distribution of mastitis pathogens from culture-positive samples over the months during the entire study period. The detection of environmental pathogens like *Sc. uberis*; other esculin-positive streptococci, including *Enterococcus* spp. and *Lactococcus* spp.; and Gram-negative pathogens, including *E. coli* and *Serratia* spp., increased during the warmer months from June to October (pooled average 37%) compared to the colder months from November to May (pooled average 32%, *p* < 0.01).

*Sc. uberis* isolates were detected at an average of 21% of culture-positive QMSs from June to October, but at an average of 18% of QMSs in the colder months of November to May. The situation was similar with other esculin-positive streptococci and Gram-negative pathogens, with an average of 2% more isolates detected in the months of June to October in each case (*p* < 0.01, respectively). In contrast, the detection of *S. aureus* fell from an average of 21% during November to May to an average of 17% during the warmer months of June to October (*p* < 0.01). Similarly, the detection of NAS fell from 31% to 29% during the same period (*p* < 0.01). *Sc. dysgalactiae* and *Sc. agalactiae* showed no differences depending on the season (on average 9% and 3%, respectively, across months).

## 4. Discussion

A strength of this study is that a large number of isolates from numerous herds and cows were included over a 10-year period. In addition, QMSs from different clinical scores were analyzed in a single milk quality laboratory. This made it possible to evaluate trends in a specific region. Enrollment of herds or submission of individual samples was based on voluntary submissions rather than a random sample. Therefore, statements regarding the prevalence of pathogens within herds of this region should be avoided.

The majority of samples stemmed from herd screening, where TGD technicians took aseptic QMSs from all four quarters of every lactating cow of the herd (or a subset of cows in larger herds). Consequently, most samples in this study came from CMT-negative or subclinically affected quarters. Accordingly, a higher proportion of QMSs from clinically affected quarters came from individual submissions compared to herd samples, as veterinarians and farmers are more likely to submit samples to base their therapy on laboratory results. Furthermore, we found that *E. coli* and *T. pyogenes* were the only pathogens that were each more often found in samples from individual cows. An important factor that influences the prevalence is the duration of the infection [19]. *E. coli* is known to cause short infections (10–30 days) and is quickly eliminated by the host’s immune response [20]. For *T. pyogenes*, Wente et al. [21] found an infection duration of one week to seven months. The timing of the sampling therefore plays an important role in the detection of mastitis pathogens, since sampling later during the infection likely leads to a no-growth result. As veterinarians and farmers often submitted acute mastitis cases, where the probability of detecting these pathogens is higher, these pathogens were detected more frequently in individual samples. When sampling entire herds at one time point, it is unlikely to catch clinical mastitis cases early during infection and therefore mastitis cases due to pathogens with a short infection period are more likely to end up in the “no-growth” category.

When interpreting our data, it should be taken into account that we only investigated the distribution of mastitis pathogens from QMSs sent to the TGD milk quality laboratory and the results may be biased by the high proportion of herd samplings and QMSs from CMT-negative and subclinically affected quarters. In our study, NAS were the most common pathogens in culture positive samples. Tenhagen et al. [22] found NAS to be the most common minor pathogens in QMSs from northern German dairy farms, and the German Veterinary Association observed the same Germany-wide [23]. Other studies also showed that NAS were most frequently detected in countries other than Germany, e.g., Norway [24], Slovakia [25], and Australia [26]. In addition, a study from Condas et al. [27] reported NAS to be the pathogens most frequently isolated from bovine milk worldwide. In most studies, NAS were the most frequently detected pathogens in quarters with subclinical mastitis [28]. This is consistent with our findings. However, we found NAS in 6% of QMSs from clinical mastitis, which is comparable to a study from Belgium, where they found NAS in 5% of all clinical samples [29], but different to a study from Finland, with 23.71% of NAS in clinical samples [30]. Furthermore, we found that the likelihood of NAS isolation increased throughout the study period, as also reported in Norway [24]. Zigo et al. [31] noted that this increase occurred after a reduction in main pathogens and that NAS are characterized by increased resistance to antibiotics and disinfectants, which may explain the rising distribution. Despite their surge, NAS are considered minor pathogens and therefore of lesser importance for dairy production [28].

The second most common pathogen in our study was *S. aureus*. We were more likely to find *S. aureus* than other German regions [22,23,32]. This may be explained by the sample selection (high number of QMSs from CMT-negative and subclinically affected quarters) and different herd structures in the regions. Fadlelmoula et al. [33] found that the risk of mastitis due to contagious pathogens is higher in small herds. Eastern German dairy herds are larger than Bavarian herds [22]. Furthermore, Wang et al. [34] noted that the prevalence of *S. aureus* varies greatly regional and worldwide. They further described that the prevalence of *S. aureus* has decreased over the last decade in China, which we also found in our study. Wang et al. [34] concluded that this development is due to rapid technological development and biosecurity measures taken by farms. Additionally, Munoz et al. [35] found that Bavarian farms have become larger and increased in their performance. In contrast, Acharya et al. [36] found an increasing proportion of *S. aureus* between 2008 and 2017 in Ontario. Similarly, Kortstegge and Krömker [32] noted an increasing risk of *S. aureus* in bulk tank milk with increasing herd sizes. Furthermore, Smistad et al. [24] observed a higher proportion of *S. aureus* (24.5%) in their study in Norway, and found *S. aureus* to be the most frequently detected major pathogen. They stated that the prevalence of *S. aureus* in Norway was relatively stable between 2000 and 2020 and explained that measures such as routine teat disinfection after milking are less consistently carried out in Norway. In addition to our results, Karell et al. [37] found an overall decreasing resistance trend in *S. aureus* in their study in Bavaria and concluded that this trend can be seen as a success of the measures taken in recent decades to tighten the use of antimicrobials and to control mastitis pathogens, which aimed to prevent new infections and eliminate existing infections. Therefore, the decreasing likelihood of detection and resistance of *S. aureus* can be seen as a success of the measures taken to combat mastitis pathogens in recent decades. Another notable observation was that *S. aureus* isolates were also fairly commonly detected in QMSs from CMT-negative and subclinically affected quarters. This is important, as Karell et al. [37] observed that *S. aureus* isolates from healthy or subclinical quarters were more likely to be in vitro resistant than isolates from clinical quarters. Woudstra et al. [9] found in their study that one *S. aureus* strain could cover 80% of the infections within a herd and that infected udder quarters are the main reservoir, underlining the contagious nature of this pathogen. Therefore, it could be concluded that cows with undetected infections act as a reservoir for within-herd transmission of *S. aureus* that also may carry virulence and resistance genes. The most important focus in preventing *S. aureus* infections is on reducing transmission from infected to uninfected quarters [9,38]. For this purpose, healthy cows or cows with subclinical mastitis should also be included in herd screenings in order to find potential sources of infection.

*Sc. uberis* was the third most common pathogen in culture positive samples in this study (19%). Its detection risk within culture-positive samples aligned with other reports from Germany (20.3%, [23]). It is noteworthy that it was the most common pathogen isolated from QMSs from clinically affected quarters (32%), which agrees with other studies from around the world [13,29,39]. In our study, *Sc. uberis* isolates showed an overall increase over the entire study period, which was also observed in Ontario by Acharya et al. [36] and previously by Phuektes et al. [40] for other parts of the world. Cobirka et al. [25] stated that *Sc. uberis* is mostly present in bedding material and that control measurements, such as post-milking teat disinfection and dry cow therapy, are far less effective against environmental pathogens, such as *Sc. uberis*. This may explain the increasing probability of *Sc. uberis* detection, since a decline in contagious pathogens (e.g., *S. aureus*), has been reported to go hand in hand with an increase in Gram-negative and therefore environmental pathogens [41]. In line with this, we found that the detection of other environmental pathogens such as *E. coli* and *Serratia* spp. also increased throughout the study period. Furthermore, the detection likelihood of *E. coli* jumped from 2018 onwards compared to previous years—especially in clinical cases. Since the number of individual submissions from clinical mastitis also increased at the same time, one can see the impact of a change in legislation in Germany in 2018 for the use of antimicrobials in veterinary medicine. It aimed to minimize the use of critically important antimicrobials (Verordnung über tierärztliche Hausapotheken, TÄHAV) [42] and included obligatory antimicrobial susceptibility testing, for example, if critically important antimicrobials were selected or antimicrobials were changed during therapy. Interestingly, this increase was especially observed for samples that tested positive for *E. coli*. This was also reported by Pirner et al. [43], who investigated the resistance of Gram-negative pathogens in Bavaria. One possible explanation could be that most of the pathogens detected in this study were Gram-positive pathogens. Those pathogens are more likely to cause subclinical mastitis and were commonly detected in herd screenings even before 2018 [43]. The change in legislation therefore did not impact their detection as much as it did for *E. coli* with its short infection duration. As QMSs now had to be sampled as soon as clinical signs appeared for treatment decisions [43], this increased the likelihood of detecting *E. coli*. *E. coli* is the most common Gram-negative pathogen to cause clinical mastitis worldwide [44]. In our study, *E. coli* was the most common Gram-negative pathogen in culture-positive QMSs from clinically affected quarters (11%). In Lower Saxony, Germany, Krebs et al. [45] reported in their study a much higher detection of *E. coli* from culture-positive clinical samples (35.2%). Other authors found *E. coli* to be associated with 19.8% of clinical mastitis cases in England and Wales [46], 15.5% in Belgium [29], or 27% of cases in China [47]. This underlines that the distribution of pathogens may vary depending on geographical region [48].

Besides region, a variety of factors can influence the prevalence of mastitis-causing pathogens, such as herd-size, housing system, and season [49]. Season also impacts cows in Germany, as they suffer from heat stress too [50]. The temperature–humidity index (THI) is widely used to assess heat stress, and a high THI is associated with an increased somatic cell score [51,52]. In our study, we found seasonal changes in the occurrence of QMSs from subclinically affected quarters, as they were slightly more common during the warmer months of June to October. Furthermore, we found differences in the detection of various mastitis pathogens depending on the month and therefore season. Environmental pathogens (e.g., *Sc. uberis*, Gram-negative as well as other esculin-positive pathogens) were more likely detected during the warmer months of June to October, while the proportion of *S. aureus* and NAS slightly decreased during that time. High temperatures and humidity may increase the probability of IMIs caused by environmental pathogens [53]. Other studies also reported that the prevalence of environmental mastitis due to, e.g., *Sc. uberis* [30,54] and *E. coli*, was the highest in summer and autumn [30], and the frequency of *S. aureus* and NAS mastitis cases was higher during winter [30,55]. All three studies noted a housing difference during the season, which may explain the observed distribution. Olde Riekerink et al. [54] stated that cows on pasture during summer are at an increased risk of environmental mastitis caused by pathogens such as *Sc. uberis.* The warm and humid conditions that prevail in summer, combined with the organic material on the pastures, create a favorable environment for these pathogens to thrive [54]. However, other studies reported an increased incidence of environmental mastitis or somatic cell count in hot and humid weather and explained this with immunosuppression due to heat stress and a higher pathogen load in the environment, leading to increased susceptibility to IMIs [56,57]. Furthermore, Hamel et al. [12] found higher shedding of *Sc. uberis* with a higher THI, which may be a possible explanation for the higher detection of *Sc. uberis* in summer. Other possible explanations are given by Kabelitz et al. [11], who suggest that high temperatures and thus the higher reproduction rate of pathogens in the environment could be a reason, as well as the transmission of bacteria by flies, which are particularly present in summer. In this study, however, we could only report the observed differences, as we did not have the climate or other risk factors like farming practices for all submissions. Therefore, we can only suggest explanations for the observed dynamics, and further research is needed. Nevertheless, increasing temperatures due to climate change, increasing heat stress, and therefore vulnerability to mastitis, as well as the influence on mastitis pathogens, likely bring new challenges for maintaining udder health [58].

Overall, the increase in environmental pathogens observed in this study emphasizes the need to continue and improve management practices. Effective control of environmental mastitis can be achieved by minimizing teat-end exposure to these pathogens and enhancing cow resistance to intramammary infections, e.g., through vaccination strategies [59]. Reducing the exposure of cows to environmental mastitis pathogens also involves maintaining clean and dry bedding, regular removal of manure, and avoiding overcrowding in barns and pastures [60]. In addition, season-dependent measures such as appropriate cooling can reduce heat stress for cows, thereby strengthening their immune response and reducing the likelihood of mastitis outbreaks [61].

## 5. Conclusions

This study underscores the dynamic nature of mastitis pathogen distribution, influenced by mastitis status and seasonal factors. Contagious pathogens such as *S. aureus* have decreased over the last decade, while environmental pathogens continue to play an important role for udder health in Bavaria. In addition, the study results emphasize that both healthy cows and those with subclinical mastitis can serve as a reservoir for mastitis pathogens. It is therefore essential to consider healthy cows and cows with subclinical mastitis as a reservoir for mastitis pathogens during monitoring and control efforts in order to track trends and adapt targeted prevention measures.

## Figures and Tables

**Table 1:** All quarter milk samples (QMSs) between 2014 and 2023. The QMSs stemmed from entire or partial herd screenings (herd) or submissions from individual cows (individual).

Year	QMS (n)	Herds (n)	Cows (n)	Herd (%)	Individual (%)	CMT ^1^ Negative (%)	Subclinical Mastitis (%)	Clinical Mastitis (%)
All	3,886,162	15,609	634,022	83	17	71	27	2
2014	382,752	4957	97,364	87	13	74	25	2
2015	371,039	4820	94,878	86	14	72	27	2
2016	389,947	4646	99,713	83	17	72	27	2
2017	401,453	4719	103,335	81	19	71	27	2
2018	451,573	5767	116,397	81	19	71	27	2
2019	395,016	4899	101,980	82	18	73	25	2
2020	380,001	4689	98,385	82	18	67	31	2
2021	388,417	4479	99,747	82	18	69	29	2
2022	350,167	3773	89,689	82	18	70	27	2
2023	375,797	3774	96,993	83	17	72	26	2

^1^ California Mastitis Test.

## Data Availability

Data are contained within the article.
